# Selected novel 5'-amino-2'-hydroxy-1, 3-diaryl-2-propen-1-ones arrest cell cycle of HCT-116 in G_0_/G_1 _ phase

**DOI:** 10.17179/excli2015-610

**Published:** 2016-01-13

**Authors:** Lalitha Simon, K. K. Srinivasan, Nitesh Kumar, Neetinkumar D. Reddy, Subhankar Biswas, C. Mallikarjuna Rao, Sudheer Moorkoth

**Affiliations:** 1 Department of Chemistry, Manipal Institute of Technology, Manipal University, Manipal, 576 104, India; 2 Department of Chemistry, Shri Madhwa Vadiraja Institute of Technology & Management, (affiliated to Visvesvaraya Technological University, Belgaum), Bantakal, Udupi, 574115, India; 3 Department of Pharmacology, Manipal College of Pharmaceutical Sciences, Manipal University, 576 104, India; 4 Department of Pharmaceutical Quality Assurance, Manipal College of Pharmaceutical Sciences, Manipal University, 576 104, India

**Keywords:** 5'-amino-2'-hydroxy-1,3-diaryl-2-propen-1-ones, cytotoxicity, MTT, acridine orange-ethidium bromide nuclear staining, flow cytometry

## Abstract

A series of 5'-amino-2'-hydroxy-1,3-diaryl-2-propen-1-ones (AC1-AC15) were synthesized by Claisen-Schmidt condensation of 5'-acetamido-2'-hydroxy acetophenone with various substituted aromatic aldehydes. The synthesized compounds were characterized by FTIR, ^1^H NMR and mass spectrometry and evaluated for their selective cytotoxicity using MTT assay on two cancer cell lines namely breast cancer cell line (MCF-7), colon cancer cell line (HCT-116) and one normal kidney epithelial cell line (Vero). Among the tested compounds, AC-10 showed maximum cytotoxic effect on MCF-7 cell line with IC_50 _value 74.7 ± 3.5 µM. On HCT-116 cells, AC-13 exhibited maximum cytotoxicity with IC_50 _value 42.1 ± 4.0 µM followed by AC-14 and AC-10 with IC_50 _values 62 ± 2.3 µM and 95.4 ± 1.7 µM respectively. All tested compounds were found to be safe on Vero cell line with IC_50_ value more than 200 µM. Based on their highest efficacy on HCT-116, AC-10, AC-13 and AC-14 were selected for mechanistic study on this cell line by evaluating changes nucleomorphological characteristics using acridine orange-ethidium bromide (AOEB) dual stain and by analyzing cell cycle with flow cytometry using propidium iodide stain. In AOEB staining, all three tested compounds showed significant (p < 0.05) increase in percentage apoptotic nuclei compared to control cells, with highest increase in apoptotic nuclei by AC-13 treatment (31 %). Flow cytometric studies showed cell cycle arrest by AC-10 and AC-14 treatment in G_0_/G_1_ phase and by AC-13 in G_0_/G_1 _and G_2_/M phase. The study reflected the potential of AC-10, AC-13 and AC-14 to be the lead molecules for further optimization.

## Introduction

Cancer is rapid and uncontrolled growth of abnormal cells. It is the major cause of death, after cardiovascular diseases all over the world (Bandgar et al., 2010[1]). Most of the drugs available today for the treatment of cancer are cytotoxic in nature, which act by interfering with the operation of the cell's DNA in some way. These cytotoxic drugs are very harmful to the body unless the drugs are specific to target cancer cells. The cancer specific targeting by cytotoxic agents is difficult to achieve because the modifications, which convert healthy cell into cancerous one are very subtle. Thus a major challenge is always present in designing new drugs with more selectivity for cancer cells.

1,3-Diaryl-2-propen-1-ones, commonly known as chalcones, belong to the flavonoid family (Bennett et al., 1980[2]; Chiaradia et al., 2008[4]; Chimenti et al., 2009[5]). Structurally, they contain an open-chain flavonoid skeleton in which two aromatic rings are linked by a three-carbon α,β-unsaturated carbonyl system. Chalcones exhibit a wide range of biological activities, which include anticancer (Chen et al., 1994[3]; Dimmock et al., 1999[6]), anti-inflammatory (Chiaradia et al., 2008[4]; Domínguez et al., 2001[7]; Fu et al., 2004[9]), antioxidant (Go et al., 2005[10]), antimicrobial, anti-tubercular (Gold and Moellering, 1996[11]; Kawabata et al., 2003[12]), antimalarial (Lust et al., 2005[17]) and anti-allergic activities (Kouskoura et al., 2008[14]).The biological activities of chalcones are considered to be due to the presence of a reactive α,β-unsaturated keto function, while the presence of a double bond allows these molecules to exist as cis or trans geometric isomers. The trans-isomer has been proven to be thermodynamically as well as biologically favourable.

Many naturally occurring chalcones with potent anticancer efficacy against a variety of cancer cell lines have been found. Licochalcone A, an oxygenated chalcone found in the roots of the Chinese liquorice (*Glycyrrhiza uralensis*), has been demonstrated to possess many bioactive properties including anti-parasitic, estrogenic, antimalarial and antitumor activities (Khatib et al., 2005[13]; Miranda et al., 2000[18]; Modzelewska et al., 2006[19]). Xanthohumol, a prenylated chalcone isolated from the hop cones (*Humuluslupulus *L.) is suggested to exhibit broad spectrum anticancer properties against different types of human cancer cells primarily through inhibition of proliferation and induction of human cancer cell apoptosis (Mosmann, 1983[20]; Nowakowska, 2007[22]). Flavokawain A, B, and C (Go et al., 2005[10]; Gold and Moellering, 1996[11]; Kawabata et al., 2003[12]) in kava extracts have been shown to possess strong antiproliferative and apoptotic effect in human bladder cancer cells (Palkar and Master, 2000[23]).

Introduction of amino groups into various heterocyclic systems had led to very effective therapeutic agents. 5-Amino flavones showed antitumor activity highly selective to the ER-positive breast cancer cell line (Pan et al., 2005[24]). 6-Aminoflavone inhibited mammalian intestinal α-glucosidase (Srinivasan et al., 2009[28]). 5,4'-Diamino-6,8,3'-trifluoroflavone (Wu et al., 2011[29]) exhibited strong growth inhibitory activity against MCF-7 cells. But very little has been reported on the synthesis and biological activities of chalcones with a free amino substitution in the ring A. Studies on the anticancer potential of chalcones having amino group in the 5^th^ position, and hydroxyl group at the 2^nd^ positions of ring A of chalcones have not been reported as of our knowledge. In view of the wide spectrum of medicinal applications of chalcone derivatives, our research continues to explore the anticancer potential of flavonoids having free amino group/s. The present study includes the synthesis, characterization and mechanistic insight into anticancer activities of some new 5'-amino-2'-hydroxy-1,3-diaryl-2-propen-1-ones. 

## Materials and Methods

### Experimental

**Figure 1:**
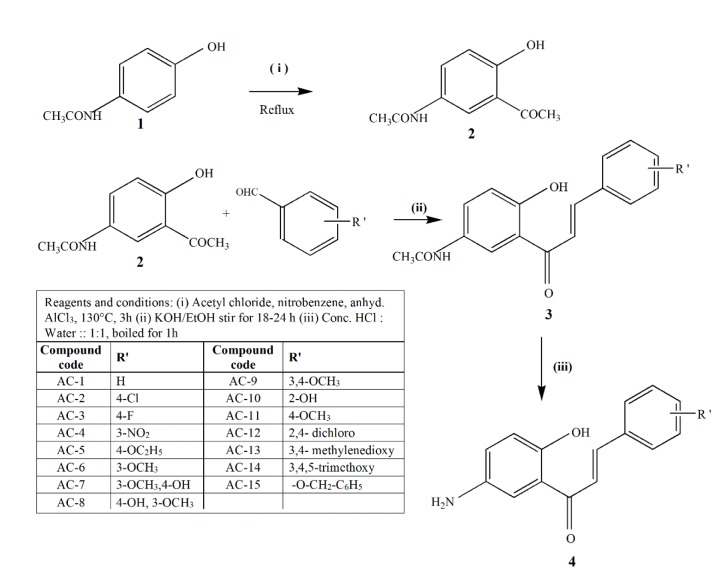
Scheme for the synthesis of 5'-amino-2'-hydroxy-1,3-diaryl-2-propen-1-ones

The chemicals required for the synthesis were purchased from Sigma Aldrich, Hi-Media, Loba Chemicals and Nice Fine Chemical India. Melting points were recorded by open capillary method and purity was assessed using Rf value in thin layer chromatography (TLC) on pre-coated silica gel aluminium backed plates (Kieselgel 60 F254 Merck (Germany)). The IR spectra in KBr pellets were recorded using Shimadzu FTIR 8400S spectrophotometer. ^1^H NMR spectra were recorded by Bruker AV400 (400MHz) spectrometer in deuterated dimethyl sulphoxide using tetramethyl silane as internal standard. Mass spectra were scanned on a Shimadzu LCMS (ESI) 2010A spectrometer. 5'-Acetamido-2'-hydroxy acetophenone (**2**) (Figure 1(Fig. 1)) was prepared according to the known procedure. 5'-acetamido-2'-hydroxy-1,3-diaryl-2-propen-1-ones (**3**) (Figure 1(Fig. 1)) were prepared by procedure given in the literature (Wu et al., 2003[30]).

### General procedure for the synthesis of 5'-amino-2'-hydroxy -1,3-diaryl-2-propen-1-ones (AC1 -AC15)

A mixture of concentrated hydrochloric acid and water (1:1) was added to 5'-acetamido-2'-hydroxy chalcone, and boiled for one hour. The reaction progress was monitored with TLC using petroleum ether-ethyl acetate solvent system in the ratio 2:3. When the reaction was complete, the reaction mixture was cooled to room temperature and ice cold water was added. The solution was basified by adding 10 % sodium bicarbonate solution. The product obtained was filtered and purified by recrystallization from ethanol to get amino chalcones (Figure 1(Fig. 1)).

### Characterization

*(E)-1-(5-Amino-2-Hydroxyphenyl)-3-phenylprop-2-en-1-one (AC-1): *Light Brown solid, yield: 72 %; m.p. 126 °C; IR (KBr) *ν*_max_/cm^−1^: 3425, 3340 (NH_2_*)*, 3565 (OH), 1690 (α,β-unsaturated C=O), 3026 (Ar-CH), 1136 (C-N ), 1510 (C=C); ^1^H NMR (400 MHz, DMSO-d_6_): δ ppm 5.43 (s,2H,NH_2_), 6.60 (d, 1H,J = 2.5 Hz), 6.71 (d, 1H, J = 15.7 Hz), 6.90 (d, 1H, J = 2.5 Hz), 7.47 (m, 3H), 7.51 ( d, 1H, J = 15.7 Hz), 7.71 (dd,1H, J = 2.5 Hz, 8.9 Hz), 7.79 (m, 2H), 8.17 (d, 1H, J = 2.5 Hz), 11.70 (s, OH); LCMS (*m/z*): 239 (M^+^).

*(E)-1-(5-Amino-2-Hydroxyphenyl)-3-(4-chlorophenyl)prop-2-en-1-one (AC-2): *Dark Brown solid, yield: 70 %; m.p. 138 °C; IR(KBr) *ν*_max_/cm^−1 ^: 3450, 3341 (NH_2_*)*, 3588 (OH), 1683 (α,β-unsaturated C=O), 3070 (Ar-CH), 1130 (C-N), 1510 (C=C); ^1^H NMR (400MHz, DMSO-d_6_): δppm 5.34 (s, 2H, NH_2_), 6.97 (d, 1H, J = 8.9 Hz), 7.24 (t, 2H,J = 8.9 Hz), 7.63 (dd, 1H, J = 2.5, 8.9 Hz), 7.70 (d, 1H,J = 15.7 Hz), 7.78 (d, 1H, J = 15.7 Hz), 7.84 (d, 2H, J = 8.9 Hz), 8.18 (d, 1H, J = 2.5 Hz), 11.81 (s, 1H,OH); LCMS (*m/z*): 273 (M^+^).

**(***E)-1-(5-Amino-2-Hydroxyphenyl)-3-(4-fluorophenyl)prop-2-en-1-one (AC-3): *Pale brown solid, yield: 68 %; m.p. 154 °C; IR(KBr) *ν*_max_/cm^−1^: 3430, 3329 (NH_2_*)*, 3572 (OH), 1690 (α,β-unsaturated C=O), 3062 (Ar -CH), 1130 (C-N), 1510 (C=C); ^1^H NMR (400MHz, DMSO-d_6_): δppm; 5.48 (s, 2H, NH_2_), 6.97 (d, 1H, J = 8.9 Hz), 7.32 (d, 2H,J = 8.9 Hz), 7.71 (dd, 1H, J = 2.5, 8.9 Hz), 7.73 (d, 1H,J = 15.7 Hz), 7.78 (d, 1H, J = 15.7 Hz), 7.90 (t, 2H, J = 8.9 Hz), 8.18 (d, 1H, J = 2.5 Hz), 11.73 (s, 1H,OH); LCMS (*m/z*): 257 (M^+^).

(*E*)-*1-(5-Amino-2-Hydroxyphenyl)-3-(3-nitrophenyl)prop-2-en-1-one (AC-4): *Brown solid, yield: 50 %; m.p. 112 °C; IR (KBr) *ν*_max_/cm^−1^: 3420, 3319 (NH_2_*)*, 3570 (OH), 1676 (α,β-unsaturated C=O) 3060 (Ar-CH), 1130 (C-N), 1510 (C=C); ^1^H NMR (400 MHz, DMSO-d_6_): δppm: 5.40 (s, 2H, NH_2_), 6.97 (d, 1H, J = 8.9 Hz), 7.46 (d, 2H,J = 8.9 Hz), 7.88 (dd, 1H, J = 2.5, 8.9 Hz), 7.93 (d, 1H,J = 16.2 Hz), 8.01 (d, 1H, J = 16.2 Hz), 8.26 (t, 2H, J = 8.9 Hz), 8.30 (d, 1H, J = 2.5 Hz), 11.86 (s,1H,OH); LCMS (*m/z*): 284 (M^+^).

(*E*)-*1-(5-Amino-2-Hydroxyphenyl)-3-(4-ethoxyphenyl)prop-2-en-1-one (AC-5): *Brown solid, yield: 68 %; m.p. 135 °C; IR(KBr) *ν*_max_/cm^−1^: 3420, 3329 (NH_2_), 3568 (OH), 1692 (α,β-unsaturated C=O), 3060 (Ar-CH), 1130 (C-N), 1510 (C=C); ^1^HNMR (400 MHz, DMSO-d_6_): δppm: 1.40 (t,3H,CH_3_), 4.23 (q,2H,OCH_2_), 5.43 (s,2H,NH_2_), 6.75 (d,1H,J=8.4Hz), 6.86 (d,1H,J=16Hz), 6.98 (d,1H,J=8.0Hz), 7.06 (d,2H,J=8.0Hz), 7.46 (d,2H,J=8.0Hz), 7.771 (d,1H,J=16Hz), 7.84 (d,1H,J=8.8Hz), 11.8 (s,1H,OH); LCMS (*m/z*): 283 (M^+^). 

(*E*)-*1-(5-Amino-2-Hydroxyphenyl)-3-(3-methoxyphenyl)prop-2-en-1-one (AC-6): *Brown solid, yield: 79 %; m.p. 110 °C; IR (KBr) *ν*_max_/cm^−1^: 3415, 3329 (NH_2_), 3588 (OH), 1672 (α,β-unsaturated C=O), 3060 (Ar -CH), 1130(C-), 1510(C=C); ^1^H NMR (400 MHz, DMSO-d_6_): δppm: 3.81 (s, OCH3), 5.43 (s,2H,NH_2_), 6.96 (d, 1H, J = 8.9 Hz), 7.04 (m,1H), 7.37 (m, 3H), 7.71 (dd, 1H, J = 8.9, 2.5 Hz), 7.72 (d, 1H, J = 15.7 Hz), 7.79 (d, 1H, J = 15.7 Hz), 8.14(d, 1H, J = 2.5 Hz, 11.71 (s, OH); LCMS (*m/z*): 269 (M^+^).

(*E*)-*1-(5-Amino-2-Hydroxyphenyl)-3-(4-hydroxy-3-methoxyphenyl)prop-2-en-1-one (AC-7): *Brown solid, yield: 61 %; m.p. 136 °C; IR (KBr) *ν*_max_/cm^−1^: 3446, 3301 (NH_2_), 3591 (OH), 1680 (α,β-unsaturated C=O), 3078 (Ar-CH), 1138 (C-N), 1520 (C=C); ^1^H NMR (400 MHz, DMSO-d_6_): δppm: 3.89 (s,3H,OCH_3_), 5.37 (s,2H,NH_2_), 7.84 (d,1H,J=8.8Hz), 7.77 (s,1H), 7.46 (d,2H,J=8.0Hz), 7.06 (d,2H,J=8.0Hz), 6.98 (d,1H,J=8.0Hz), 6.86 (d,1H,J=16Hz), 6.75 (d,1H,J=8.4Hz), 11.8 (s,1H,OH); LCMS (*m/z*): 285 (M^+^).

(*E*)-*1-(5-Amino-2-Hydroxyphenyl)-3-(3-hydroxy-4-methoxyphenyl)prop-2-en-1-one (AC-8)****: ***Pale brown solid, yield: 61 %; m.p. 136 °C; IR (KBr) *ν*_max_/cm^−1^: 3446, 3301 (NH_2_), 3589 (OH), 1680 (α,β-unsaturated C=O), 3078 (Ar-CH), 1138 (C-N), 1520 (C=C); ^1^H NMR (400 MHz, DMSO-d_6_): δppm: 3.81 (s,3H,OCH_3_), 5.40 (s,2H,NH_2_), 7.84 (d,1H,J=8.8Hz), 7.77 (s,1H), 7.46 (d,2H,J=8.0Hz), 7.06 (d,2H,J=8.0Hz), 6.98 (d,1H,J=8.0Hz), 6.86 (d,1H,J=16Hz), 6.75 (d,1H,J=8.4Hz), 11.5 (s,1H,OH); LCMS (*m/z*): 285 (M^+^).

(*E*)-*1-(5-Amino-2-Hydroxyphenyl)-3-(3,4-dimethoxyphenyl)prop-2-en-1-one (AC-9): *Brown solid, yield: 73 %; m.p. 90 °C; IR(KBr) *ν*_max_/cm^−1^: 3430, 3329 (NH_2_), 3588 (OH), 1690 (α,β-unsaturated C=O), 3062 (Ar-CH), 1130 (C-N), 1510 (C=C); ^1^H NMR (400 MHz, DMSO-d_6_): δppm: 3.78 (6H,s, 23 x OCH_3_), 5.48 (s, 2H, NH_2_), 6.97 (d, 1H, J = 8.9 Hz), 7.32 (d, 2H, J = 8.9 Hz), 7.71 (d, 1H, J = 2.5, 8.9 Hz), 7.73 (d, 1H,J = 15.7 Hz), 7.78 (d, 1H, J = 15.7 Hz), 7.90 (s, 1H), 8.18 (d, 1H, J = 2.5 Hz), 11.73 (s, 1H,OH); LCMS (*m/z*): 299 (M^+^).

(*E*)-*1-(5-Amino-2-Hydroxyphenyl)-3-(2-hydroxyphenyl)prop-2-en-1-one (AC-10): *Dark brown solid, yield: 68 %; m.p. 130-132 °C; IR(KBr) *ν*_max_/cm^−1^: 3448, 3320 (NH_2_), 3576 (OH), 1692 (α,β-unsaturated C=O), 3062 (Ar -CH), 1138 (C-N), 1510 (C=C); ^1^H NMR (400MHz, DMSO-d_6_): δppm.; 5.39 (s, 2H, NH_2_), 6.97 (d, 1H, J = 8.9 Hz), 7.32 d, 2H,J = 8.9 Hz), 7.64 (d, 1H, J = 2.5, 8.9 Hz), 7.75 (d, 1H,J = 15.7 Hz), 7.78 (d, 1H, J = 15.7 Hz), 7.90 (s, 1H), 8.18 (d, 1H, J = 2.5 Hz), 11.80 (s, 1H,OH); LCMS (*m/z*): 255 (M^+^).

*(E)-1-(5-Amino-2-Hydroxyphenyl)-3-(4-methoxyphenyl)prop-2-en-1-one (AC-11): *Brown solid, yield: 72 %; m.p. 102-104 °C; IR (KBr) *ν*_max_/cm^−1^: 3450, 3344 (NH_2_), 3591(OH), 1678 (α,β-unsaturated C=O), 3070 (Ar-CH), 1138 (C-N), 1515 (C=C); ^1^H NMR (400 MHz, DMSO-d_6_): δppm: 3.84 (s,3H, OCH_3_), 5.43 (s,2H,NH_2_), 7.84 (d,1H,J=8.8Hz), 7.771 (d,1H,J=16Hz), 7.46 (d,2H,J=8.0Hz), 7.06 (d,2H,J=8.0Hz), 6.98 (d,1H,J=8.0Hz), 6.86 (d,1H,J=16Hz), 6.75 (d,1H,J=8.4Hz), 11.8 (s,1H,OH); LCMS (*m/z*): 269(M^+^).

(*E*)-*1-(5-Amino-2-Hydroxyphenyl)-3-(3,4-dichlorophenyl)prop-2-en-1-one (AC-12): *Brown solid, yield: 70 %; m.p. 138 °C; IR(KBr) *ν*_max_/cm^−1^: 3450, 3341 (NH_2_), 3580 (OH), 1690 (α,β-unsaturated C=O), 3070 (Ar-CH), 1130 (C-N), 1510 (C=C); ^1^H NMR (400MHz, DMSO-d_6_): δppm: 5.34 (s, 2H, NH_2_), 7.01 (d, 1H, J = 8.9 Hz), 7.24 (d, 2H,J = 8.9 Hz), 7.63 (d, 1H, J = 2.5, 8.9 Hz), 7.70 (d, 1H,J = 15.7 Hz), 7.78 (d, 1H, J = 15.7 Hz), 7.86 (s,1H Hz), 8.18 (d, 1H, J = 2.5 Hz), 11.75 (s, 1H,OH); LCMS (*m/z*): 308 (M^+^).

(*E*)-*1-(5-Amino-2-Hydroxyphenyl)-3-(3,4-methylenedioxyphenyl)prop-2-en-1-one (AC-13): *Pale brown solid, yield: 81 %; m.p. 128 °C; IR (KBr) *ν*_max_/cm^−1^: 3446, 3301 (NH_2_*)*, 3591 (OH), 1680 (α,β-unsaturated C=O), 3078 (Ar-CH), 1138 (C-N), 1520 (C=C); ^1^H NMR (400 MHz, DMSO-d_6_): δppm: 5.40 (s,2H,NH_2_), 6.034 (s,2H,CH_2_-O), 6.90 (d,1H,J=8.4Hz), 6.96 (d,1H,J=16Hz), 6.99 (d,1H,J=8.0Hz), 7.06 (d,2H,J=8.0Hz), 7.46 (d,2H,J=8.0Hz), 7.70 (s,1H), 7.84 (d,1H,J=8.8 Hz), 11.5 (s,1H,OH); LCMS (*m/z*): 283 (M^+^).

(*E*)-*1-(5-Amino-2-Hydroxyphenyl)-3-(3,4,5-trimethoxyphenyl)prop-2-en-1-one(AC-14): *Light Brown solid, yield: 81 %; m.p. 136-138 °C; IR (KBr) *ν*_max_/cm^−1^: 3430, 3329 (NH_2_), 3588 (OH), 1690 (α,β-unsaturated C=O), 3062 (Ar-CH), 1130 (C-N), 1510 (C=C); ^1^H NMR (400 MHz, DMSO-d_6_): δppm: 3.85 (9H,s, 3 x OCH_3_), 5.48 (s, 2H, NH_2_), 7.50 (d, 1H, J = 8.9 Hz), 7.32 (d, 1H,J = 8.9 Hz), 7.71 (d, 1H, J = 2.5, 8.9 Hz), 7.73 (d, 1H,J = 15.7 Hz), 7.78 (d, 1H, J = 15.7 Hz), 7.93 (s, 2H), 11.73 (s,1H,OH); LCMS (*m/z*): 329 (M^+^).

(*E*)-*1-(5-Amino-2-Hydroxyphenyl)-3-(4-benzyloxyphenyl)prop-2-en-1-one (AC-15): *Dark Brown solid, yield: 73 %; m.p. 136-138 °C; IR (KBr) *ν*_max_/cm^−1^: 3425, 3340 (NH_2_*)*, 3565 (OH), 1690 (α,β-unsaturated C=O), 3026 (Ar-CH), 1136 (C-N), 1510 (C=C); ^1^H NMR (400 MHz, DMSO-d_6_): δppm: 5.25 (s, 2H, Ar-CH_2_-), 5.50 (s,2H,NH_2_), 7.11 (dd, 1H, J =2.5Hz, 8.9 Hz), 7.29 (d, 1H,J=17Hz), 7.47 (dd, 2H, J =2.5Hz, 8.9 Hz), 7.71 (d, 2H, J = 2.5 Hz), 7.77 (d, 2H, J = 2.5 Hz), 7.79 (d,2H, J = 2.5 Hz), 7.82 (d,1H, J = 2.5 Hz), 7.87 (d,1H,J=17Hz), 8.01 (d,1H, J = 2.5 Hz), 8.17 (d, 1H, J = 2.5 Hz), 11.70 (s, OH); LCMS (*m/z*): 345 (M^+^).

### Evaluation of cytotoxicity by MTT assay

Cytotoxicity of the synthesized compounds was evaluated in cancer cells namely human colon cancer (HCT-116) cells and human breast cancer (MCF-7) cells and normal kidney epithelial cells (Vero). These cells were originally procured from National Cancer Center for Cell Science, Pune, India and cultured in our lab using Dulbecco's modified Eagles medium (DMEM) containing 10 % fetal bovine serum (FBS) at 37 °C in an atmosphere containing 5 % CO_2_. All other chemicals used in this study were purchased from Sigma-Aldrich, USA.

Cell suspension containing 1 x 10^4^ cells in 0.1 mL was seeded in a 96 well plate for 24 h. Test compounds were serially diluted with the medium to get a stock solution of 400 µM, 200 µM, 100 µM, 50 µM. After 24 h of incubation, 100 µL of test solution from respective stocks was added in triplicate and incubated for 48 h. After the treatment, drug-containing media was removed and 20 µL of MTT reagent (5 mg/mL in PBS) was added to each well. After 4 h of incubation at 37 °C, the MTT reagent was removed and 100 µL of 100 % DMSO was added to each well to solubilize formazan crystals. The optical density was measured using an ELISA plate reader at 540 nm and percentage cytotoxicity was calculated. (Kumar et al., 2012[15]; Zi and Simoneau, 2005[31]).

### Acridine orange (AO) and Ethidium bromide double staining using fluorescent microscopy

DNA-binding dyes Acridine orange (AO) and Ethidium bromide (EB) (Sigma, USA) were used for the morphological detection of apoptotic cells. 5 x 10^3^ cells were seeded per well in 24-well plates with DMEM, containing 10 % FBS. After 24 h, cells were treated with selected concentrations of test compounds and incubated for 24 h. The media was removed and plate was washed with phosphate buffer saline (PBS, pH 7.4). Cells were fixed in ice-cold methanol for 20 min, washed with PBS again and stained with acridine orange and ethidium bromide stain (20/30 µg/ml). After incubation for 20 min at 37 °C, and washing with PBS thrice, the plate was observed under a fluorescent microscope for morphological changes in nucleus such as condensed chromatin and fragmented nuclei. The apoptotic index (AI) was calculated as % of apoptotic cells from randomly counted 100 cells in each treatment group (Reddy et al., 2015[25]).

### Cell cycle analysis

Flow cytometric analysis technique evaluates the effect of test compounds on cell cycle progression and check-points. Cells (1 x 10^6^) were seeded in 25 cm^2^ flasks and, after overnight adherence, incubated with test compounds. Then cells were detached by trypsinization and mixed with floating cells, centrifuged and washed with PBS. The cell pellets were fixed in 70 % ice-cold methanol and stored at -20 °C for 24 h. After that cell pellets were washed with PBS and isotonic PI solution [25 µM propidium iodide, 0.03 % NP-40 and 40 µg /ml RNase A] was added. The stained cells were analyzed using Accuri C6 flow cytometer (BD Biosciences, San Jose, CA, USA) with excitation at 488 nm and emission at 575/40 nm (Reddy et al., 2015[25]).

### Statistical analysis

Statistical analysis of data were performed by one way ANOVA followed by Tukey's post hoc using Graph Pad Prism v 5.03 (demo version), CA, USA, where p<0.05 was considered to be significant.

## Results

### Chemistry

**Figure 2:**
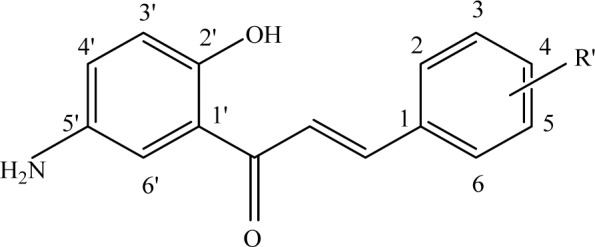
Structure of 5'-amino-2'-hydroxy chalcone

A series of chalcones having amino substituent at the 5^th^ position and hydroxyl substituent at the 2^nd^ position of ring A and different substituents in the 2^nd^, 3^rd^, 4^th^, 5^th^ positions of ring B were synthesized. The acetamido chalcones were synthesized by Claisen-Schmidt condensation of substituted benzaldehydes and 5'-acetamido-2'-hydroxy acetophenone. All the compounds were obtained in excellent yields (70-90 %). The acetamido chalcones were hydrolysed in acidic medium to obtain amino chalcones in good yields (50-81 %) (Figure 2(Fig. 2)).

### In vitro cytotoxicity by MTT assay method

Fifteen 5'-amino-2'-hydroxy chalcones were evaluated for their cytotoxicity against two human tumor cell lines. Among the compounds analyzed, seven (viz., AC-2, AC-3, AC-8, AC-9, AC-10, AC-13 and AC-14) displayed high cytotoxicity (close to 200 µM including SEM) against HCT-116 and five compounds (AC-8, AC-9, AC-10, AC-13, AC-14) against MCF-7 cells. Most of the compounds were free from cytotoxicity to normal kidney epithelial cells (Vero) at < 200 µM concentration, which indicated the selectivity of the compounds towards tested tumor cells. The results are presented in Table 1(Tab. 1). 

### AO/EB (dual) nuclear staining

**Figure 3:**
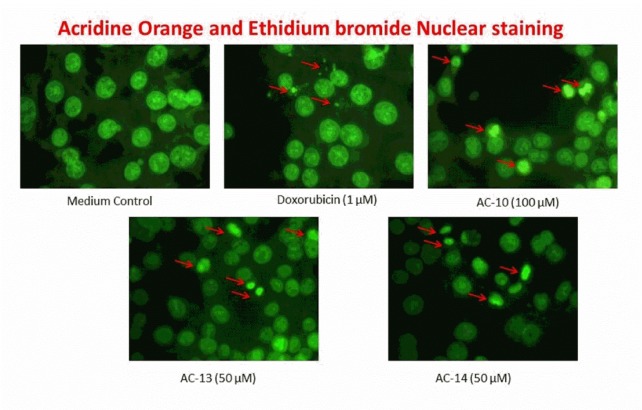
Nuclear staining. The representative images for induction of apoptosis by different treatments for 48 h in HCT-116 cells by AO/EB. Apoptotic index was calculated by counting specific pattern of condensed and fragmented nuclear morphology.

**Figure 4:**
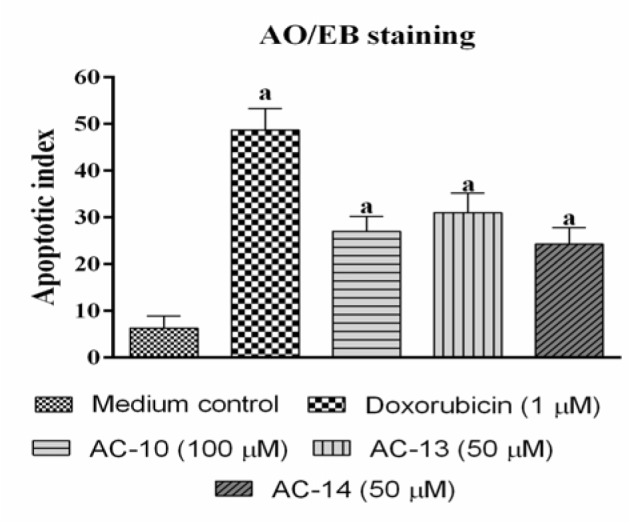
Apoptotic index. All values are mean ± SEM of three readings in triplicate. Data are analysed by one way ANOVA followed by Tukey's post hoc test, where ^a^p<0.05 compared to medium control

HCT-116 cells treated with compounds show changes in cellular morphology, including chromatin condensation and fragmented nuclei, which are characteristic features of an early apoptotic cell death (green colour) but not necrosis (orange colour) (Nayak et al., 2013[21]; Reddy et al., 2015[25]). AC-10, AC-13 and AC-14 were tested at their IC_50_ value *i.e,* 100, 50 and 50 µM, respectively. All tested compounds produced morphological changes in the nuclei and showed significant (p < 0.05) increase in apoptotic nuclei. AC-13 showed maximum increase in percentage apoptotic nuclei (31 ± 4.16 %) followed by AC-10 (27 ± 3.21 %) and AC-14 (24.3 ± 3.53 %) (Figures 3(Fig. 3) and 4(Fig. 4)).

### Cell cycle analysis

**Figure 5:**
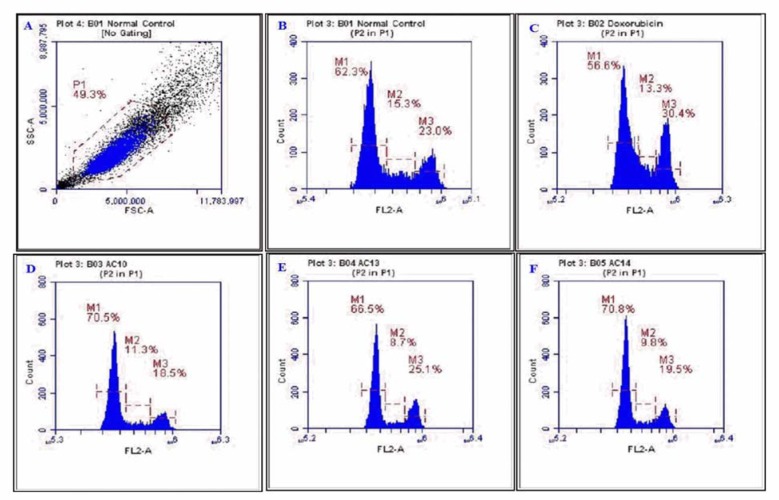
Effect on the cell cycle of HCT-116 after 48 h treatment with AC-10, AC-13 and AC-14. The concentrations used for cell cycle analysis were 100 µM for AC-10, 50 µM for AC-13 and AC-14.

The effect of the compounds on cell cycle phase was assessed and the results are shown as % cells in G_0_/G_1_, S, and G_2_/M phase. The normal control showed 62.3, 15.3 and 23.0 % cells in G_0_/G_1_, S and G_2_/M phase, respectively. The standard, doxorubicin (1 µM), accumulated 7.4 % cells in G_2_/M phase as compared with that of normal control suggesting cell cycle arrest at G_2_/M phase. The test compounds AC-10 (100 µM), AC-13 (50 µM), and AC-14 (50 µM), caused accumulation of cells (70.5 %, 66.5 % and 70.8 %), in G_0_/G_1_, phase which indicated the arrest of cell cycle in this phase. In addition AC-13 also showed accumulation of cells in G_2_/M phase (25.1 %) (Figure 5(Fig. 5)).

## Discussion

Several reports are available which state that chalcone, both derived from nature and synthetic sources, exhibit cytotoxic and antitumor activities (Ducki et al., 1998[8]). Flavokawain B, a chalcone of plant origin significantly prevents colon cancer cells growth. It produces ROS generation and GADD153 up-regulation leading to mitochondria-dependent apoptosis through release of cytochrome C and the translocation of Bak (Kuo et al., 2010[16]).

The structures of synthesized compounds were confirmed with FT-IR and ^1^H NMR and mass spectroscopy. The FT-IR spectra of the newly synthesized chalcones displayed a strong absorption band due to α,β-unsaturated C=O stretching at 1692-1672 cm^-1^, 3591-3565 cm^-1^ (Ar-OH), 3078-3026 cm^-1^ (Ar-H), 3344-3319 cm^-1^ (NH_2_). Inspection of the ^1^H-NMR spectra suggested that the chalcones presented *trans *configurations (*J *= 15-17 Hz). The other expected peaks were in the region of 11.50-11.81 ppm (s, Ar-OH), 5.34-5.50 ppm (s, Ar-NH_2_), 6.90-8.18 ppm (Ar-H). The OCH_3 _protons resonated at 3.85 ppm and were observed as a singlet. The mass spectra of all newly synthesized amino chalcones showed molecular ion peaks, which were in accordance with their respective molecular masses.

The results revealed that the most potent compounds were AC-13, AC-10 and AC-14. The 3,4-methylene substitution (AC-13), 2-hydroxy substitution (AC-10), and 3,4,5-trimethoxy substitution (AC-14) on ring B of the chalcone increase the cytotoxic activity. Regarding the structure activity relationship, it appeared that the number of methoxy groups in ring B is important for cytotoxicity. AC-14 with three methoxy groups at 3,4 and 5 positions of ring B showed good activity with IC_50_ 62 ± 2.3 µM and 196.6 ± 7.9 µM against HCT-116 and MCF-7 respectively whereas compounds having one methoxy group at 3 or 4 positions of ring B (AC-6, AC-11) and two methoxy substituents at 3 and 4 positions of ring B (AC-1) were inactive. It is observed that AC-9 with no substituent on ring B was more active than compounds having electron withdrawing groups like NO_2_, F or Cl at the 4 position of ring B. The compound AC-8 with OH substitution at 3 and OMe substitution at 4 positions of ring B showed significant cytotoxicity. However, when the positions of OH and OMe were interchanged, as in AC-7, the cytotoxicity was reduced.

Nuclear staining method using fluorescent dye is an ideal method for detection of morphonuclear changes viz., nuclear condensation, fragmentation and a disrupted membrane with cytoplasmic disintegration (Reddy et al., 2015[25]). AO/EB stain can easily permeate to the cell and stain the DNA/RNA. It is also helpful in identifying apoptotic changes in the cell. Compound with lowest IC_50_ values namely, AC-10, AC-13 and AC-14, were selected for evaluation of apoptotic changes on the colon cancer cells (HCT-116) using acridine orange/ethidium bromide double staining technique. Treatments showed increased apoptosis compared to untreated control cells. The nucleomorphological changes such as apoptotic nuclei in the form of nuclear condensation and fragmentation were significantly (p<0.05) high in the treated cells compared to control cells. AC-13 treatment was found to be more effective with about five times increase in apoptotic nuclei compared to medium control (Figures 3(Fig. 3) and 4(Fig. 4)).

Flow cytometry analysis is one of the important methods to identify the effect of cytotoxic drug on cell cycle analysis (Riccardi and Nicoletti, 2006[26]). The healthy cells undergo mitotic phase and divide into two distinct daughter cells. The cells pass through different phases of cycle namely G_0_/G_1_, S and G_2_/M to ensure proper division of the cells. Any abnormality detected in the cell cycle leads to arrest further growth with the help of check-points. In the present study, the regulation of cell cycle check-points in HCT cancer cells was studied by cell cycle analysis using propidium iodide staining. The effect of the compounds on cell cycle phase was assessed and the results of the same were shown as the percentage of cells in G_0_/G_1_, S, and G_2_/M phase. The standard, doxorubicin (1 µM), showed 7.4 % more accumulation of cells in G_2_/M phase as compared to normal control. This suggests that cell cycle arrest is at G_2_/M phase. The test compounds, AC-10 and AC-14, caused accumulation of cells in G_0_/G_1 _phase while AC-13 produced accumulation of cells in G_0_/G_1 _and G_2_/M phase. These accumulations of cells indicated the arrest of cell cycle by AC-10, and AC-14 in G_0_/G_1 _phase and by AC-13 in G_0_/G_1 _and G_2_/M phase. The results obtained were in accordance with the earlier reports on chalcones, which state that cell cycle arrest by chalcones happens in either G_0_/G_1_ phase or G_2_/M phase (Shen et al., 2007[27]).

## Conclusion

In this study, a series of fifteen 5'-amino-2'-hydroxy-1,3-diaryl-2-propen-1-ones were synthesized and characterized. The compound AC-13 exhibited maximum cytotoxicity in HCT-116 and moderate cytotoxicity in MCF-7. AC-10 exhibited best cytotoxicity in MCF-7. The AO/EB staining showed the extent of apoptotic damage. AC-13 displayed highest % of apoptotic nuclei and arrested cell cycle in G_0_/G_1 _and G_2_/M phase and AC-10 and AC-14 arrested G_0_/G_1 _phase of the cell cycle.

## Acknowledgement

The authors acknowledge Manipal Institute of Technology and Manipal College of Pharmaceutical Sciences for providing research facilities. We thank Dr. N. Gopalan Kutty, Professor, Department of Pharmacology, MCOPS, Manipal University Manipal, for his support in drafting manuscript. 

## Figures and Tables

**Table 1:**
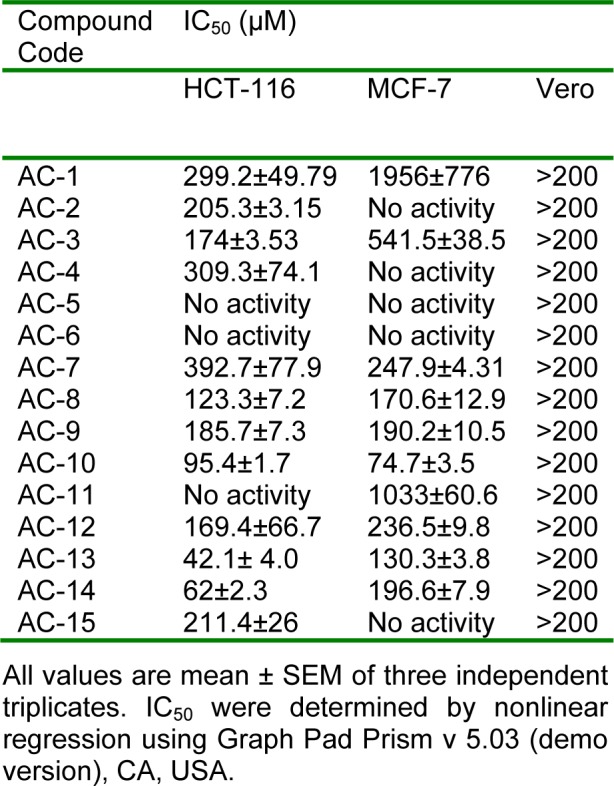
Cytotoxicity of 5'-amino-2'-hydroxy chalcones by MTT assay after 48 h incubation
